# The Role of *Helicobacter pylori* and Metabolic Syndrome-Related Mast Cell Activation Pathologies and Their Potential Impact on Pregnancy and Neonatal Outcomes

**DOI:** 10.3390/jcm13082360

**Published:** 2024-04-18

**Authors:** Maria Tzitiridou-Chatzopoulou, Evangelos Kazakos, Eirini Orovou, Paraskevi Eva Andronikidi, Foteini Kyrailidi, Maria C. Mouratidou, Georgios Iatrakis, Jannis Kountouras

**Affiliations:** 1 School of Health Sciences, Department of Midwifery, University of Western Macedonia, 50100 Koila, Greece; mtzitiridou@uowm.gr (M.T.-C.); ekazakos@gmail.com (E.K.); eorovou@uowm.gr (E.O.); 2 Second Medical Clinic, School of Medicine, Aristotle University of Thessaloniki, Ippokration Hospital, Macedonia, 54642 Thessaloniki, Greece; foteini.kyrailidi@gmail.com (F.K.); marysia.mouratidou@gmail.com (M.C.M.); 3 Department of Nephrology, Aretaieion University Hospital, School of Medicine, National and Kapodistrian University of Athens, 11528 Athens, Greece; eva_andr@hotmail.com; 4 Department of Midwifery, University of West Attica, 12243 Athens, Greece; giatrakis@uniwa.gr

**Keywords:** *Helicobacter pylori* infection, metabolic syndrome, mast cell, pregnancy outcomes, neonate outcomes

## Abstract

*Helicobacter pylori* infection, a significant global burden beyond the gastrointestinal tract, has long been implicated in various systemic pathologies. Rising evidence suggests that the bacterium’s intricate relationship with the immune system and its potential to induce chronic inflammation impact diverse pathophysiological processes in pregnant women that may in turn affect the incidence of several adverse pregnancy and neonate outcomes. *Helicobacter pylori* infection, which has been linked to metabolic syndrome and other disorders by provoking pericyte dysfunction, hyperhomocysteinemia, galectin-3, atrial fibrillation, gut dysbiosis, and mast cell activation pathologies, may also contribute to adverse pregnancy and neonatal outcomes. Together with increasing our biological understanding of the individual and collective involvement of *Helicobacter pylori* infection-related metabolic syndrome and concurrent activation of mast cells in maternal, fetus, and neonatal health outcomes, the present narrative review may foster related research endeavors to offer novel therapeutic approaches and informed clinical practice interventions to mitigate relevant risks of this critical topic among pregnant women and their offspring.

## 1. Introduction

*Helicobacter pylori* (*H. pylori*) infection represents a significant global health concern [1] and has recently received increased consideration for its potential role in various systemic pathologies [2,3,4,5]. While this microorganism is predominantly related to gastrointestinal pathologies such as gastroduodenal ulcer disease, gastric cancer, or MALT (mucosa-associated lymphoid tissue) lymphoma, increasing evidence indicates that *H. pylori* is also linked to systemic disorders [5], including adverse pregnancy and neonatal outcomes.

Recent studies show that this bacterium is associated not only with gastrointestinal disorders but also with pregnancy-related severe nausea and vomiting, hyperemesis gravidarum, metabolic disturbances, and adverse pregnancy and neonatal outcomes, including preterm labor and delivery. Moreover, the combination of these conditions further amplifies the risk in pregnant individuals [6,7]. Its presence in the maternal gut can lead to increased levels of systemic inflammation, which is considered to impact the health and development of the fetus [8]. *H. pylori* infection, beyond severe nausea and vomiting of pregnancy, is also associated with a significantly high frequency of preeclampsia, fetal growth restriction, and gestational diabetes mellitus, emphasizing the importance of screening and treating females for this bacterium prior to and throughout pregnancy to mitigate related complications [9].

Likewise, metabolic syndrome (MetS) poses a significant worldwide health issue that is increasing at an alarming rate. It includes a variety of metabolic risk factors that lead to a clinical syndrome. MetS typically includes abdominal obesity, insulin resistance (IR), type 2 diabetes mellitus (T2DM), dyslipidemia, nonalcoholic fatty liver disease (NAFLD), arterial hypertension (AH), and cardiovascular disease (CVD) [10]. Emerging evidence highlights a strong connection between MetS and active *H. pylori* infections, with both disorders appearing to mutually influence their pathophysiology [11]. This infection is associated with MetS [12], and its eradication positively impacts MetS components [13]. Furthermore, *H. pylori* is a risk factor for CVD, and its eradication is considered safe from a cardiac standpoint [14]. Recent meta-analysis underscores a correlation between *H. pylori* infection and both MetS and IR [15]. Scientific findings demonstrate that *H. pylori* infection independently contributes to MetS-related nonalcoholic fatty liver disease (NAFLD), now known as metabolic dysfunction-associated fatty liver disease (MAFLD) or metabolic dysfunction-associated steatotic liver disease (MASLD) [16], correlating with an increased degree of steatosis [17]. *H. pylori* infection also independently correlates with the severity of MetS-related non-alcoholic steatohepatitis (NASH), IR, dyslipidemia, and AH [18]. This relationship also spreads to pregnancy conditions [19].

Specifically, MetS-related parameters, IR, dyslipidemia, and AH are contributors to systemic pathologies [18], including negative pregnancy and neonatal outcomes like heightened preeclampsia risk, altered fetal growth, and premature birth [20,21,22]. MAFLD, as the hepatic component of MetS, is associated with a high risk of adverse outcomes for both the mother and the fetus [23,24]. Moreover, MetS can lead to serious complications during pregnancy, such as gestational diabetes, AH, and preeclampsia. The effects of MetS on pregnancy are significant; it impacts maternal health and has enduring consequences for the child, potentially leading to metabolic disorders later in life.

Active *H. pylori* infection is further implicated in additional MetS-related systemic disorders, particularly cardio-cerebrovascular diseases (C-CVD) and neurodegenerative disorders, which represent ultimate outcomes of MetS [3,25].

Mast cells (MCs), initially recognized for their role in allergic and anaphylactic responses, are essential effectors in the innate immune system and play pivotal roles in both innate immune responses and the regulation of adaptive immunity [26]. Recent studies have shown their participation in MetS-related disorders like MAFLD and its systemic complications [26] and adverse outcomes for both mother and fetus [23].

MC activation holds a complex position during pregnancy. While necessary for normal immune function, overactivation can lead to excessive inflammation, adversely affecting both mother and fetus. This concern is amplified in the occurrence of *H. pylori* infection and MetS, where an already elevated inflammatory state may be further intensified. In this context, MC activation may contribute to the pathophysiology of preeclampsia [8] and may have harmful effects throughout pregnancy and the post-partum period [27]. Additionally, MCs are related to dyslipidemia, atherosclerosis, and AH [28], conditions that pose risks for systemic pathologies [29], including adverse pregnancy and neonatal outcomes [30,31].

The interactions between *H. pylori*, MetS, and MC activation during pregnancy have been studied, but their combined impact on pregnancy remains understudied. *H. pylori* infection may exacerbate the inflammatory state caused by MetS and, together, they could amplify MC activation. This cascade of events is hypothesized to contribute to adverse pregnancy and neonatal outcomes, such as an augmented risk of miscarriage, preterm birth, or low birth weight, among others. Despite these considerations, searches using keywords like “*H. pylori*, metabolic syndrome, mast cell activation, pregnancy outcomes” in the international database PubMed yield no relevant research results. Therefore, this narrative review aims to examine the possible effect of combined *H. pylori* and MetS on MC activation-related pregnancy and neonatal outcomes. This review underscores the urgent need for further investigation and presents opportunities for the introduction of novel therapeutic strategies to address this crucial issue.

### 1.1. Potential Impact of H. pylori/MetS on Pregnancy and Neonatal Outcomes

The global prevalence of *H. pylori* infection ranges from 50 to 58%, partly due to migration from countries with a high prevalence of the infection, accounting for approximately 4.4 billion infected individuals [3,32]. Additionally, combined prevalence figures for MetS stand at 24%, with its individual components such as overweight and obesity varying between 35.6 and 44.1% [33,34].

Growing evidence supports the potential link of this infection with MetS-associated systemic disorders [2], such as T2DM, dyslipidemia, AH, MAFLD, C-CVD, and neurological pathologies [3,5,29].

Especially, *H. pylori* infection is implicated in IR, a vital element of MetS that is crucial for the pathophysiology of atherosclerosis and damage to target organs caused by AH. Beyond the aforementioned pathologies, current *H. pylori* infection-related MetS seems to influence critical pregnancy and neonatal outcomes, and eradication could benefit pregnant women and their newborns. Hence, additional research is warranted [35].

#### 1.1.1. The Role of *H. pylori*/MetS-Related Pericyte Dysfunction Pathologies and Their Potential Impact on Pregnancy and Neonatal Outcomes

Pericytes, which are implicated in *H. pylori*/MetS-related AH and Τ2DM pathophysiology [36,37], are specialized cells closely associated with the vasculature system, playing a key role in regulating endothelial cell characteristics and ensuring the constancy and preservation of blood vessels essential for normal vascular functionality [38]. The collaboration between endothelium and pericytes is critical for appropriate microvascular development, constancy, and maintenance [39,40]. For instance, cerebral pericytes are crucial in the neurovascular unit, managing cerebral blood flow and sustaining the integrity of the blood–brain barrier (BBB). They encase endothelial cells at the capillary level, strategically positioned to regulate and preserve the BBB [41,42]. A deficiency in cerebral pericytes within the murine brain can lead to BBB disruption, harmful leakage of circulated proteins, microvascular regression, and cerebral hypoxia [41,42]. These dynamics may collaboratively impact the neuronal interface, causing neurodegeneration, as seen in pericyte-deficient mice model [42,43].

Beyond the elements of MetS-related pericyte deficits [44] and potential cerebral pericyte dysfunction related to *H. pylori* infection [45], a deficiency in cerebral pericytes linked to MetS may play a role in the pathophysiology of neurodegenerative disorders associated with *H. pylori* and MetS [4,46], such as Alzheimer’s disease (AD) [47]. Pericyte loss is implicated in the pathophysiology of diverse MetS-associated pathologies, including T2DM, stroke, and AD. Cerebral complications of T2DM are marked by pericyte loss, augmented BBB dysfunction, and neuronal damage [48]. Notably, pericyte loss is observed in diverse areas of the brain of AD patients [49,50].

**Figure 1:**
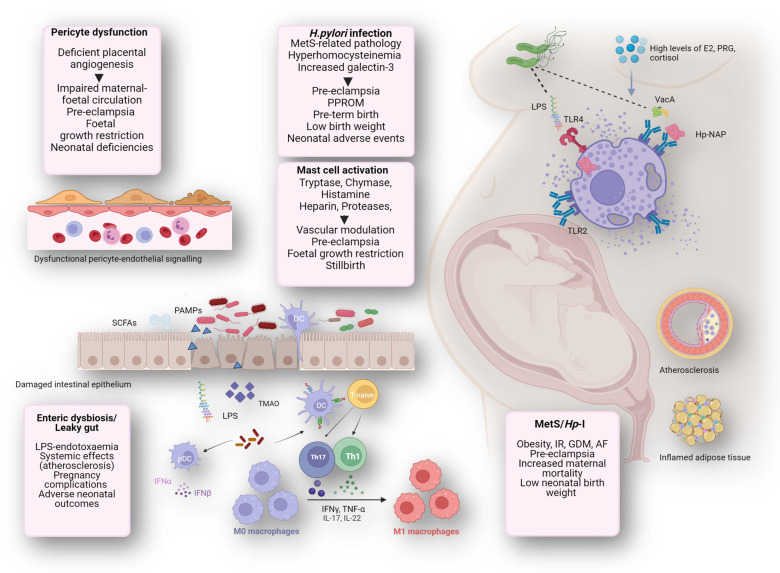
Contribution of *H. pylori*/MetS-connected effector mechanisms to mast cell activation pathophysiology and its impact to adverse pregnancy and neonatal outcomes.

Recent evidence suggests a potential link between pregnancy and maternal cardiac dysfunction, as well as alterations in placental pericytes [51]. In pregnancies affected by gestational T2DM, there may be a loss of pericyte function, characterized by increased vascular permeability and junctional disruption [51,52]. Since endothelial cells and pericytes play crucial roles in regulating angiogenesis during later stages of pregnancy, dysfunctional signaling between these cells could contribute to the development of placental vasculopathies, such as those observed in preeclampsia. Moreover, dysregulation of endothelial/pericyte signaling during the early stages of placental vasculogenesis might also be involved in endothelial dysfunction associated with preeclampsia, as well as other complications like fetal growth restriction or neonatal deficiencies (Figure 1). Further research is required to thoroughly investigate these potential links.

During pregnancy, hormonal changes may directly influence mast cell proliferation and stimulation, promoting angiogenesis, remodeling, and spiral artery modifications necessary for successful implantation and subsequent placentation into the endometrium, albeit at the expense of increased myometrium contractility. However, mast cell overactivation can lead to excessive inflammation, potentially harming the mother and fetus. The concurrent presence of *H. pylori* infection/MetS-related pathologies may dysregulate mast cell activation, shifting the immunological response towards a proinflammatory state associated with various adverse pregnancy and neonatal outcomes.

*H. pylori* infection/MetS, by provoking pericyte dysfunction, hyperhomocysteinemia, galectin-3, atrial fibrillation, gut dysbiosis, and mast cell activation pathologies, may contribute to adverse pregnancy and neonatal outcomes. Excessive secretion of mast cell mediators, including histamine, might undermine maternal vasculature adaptations, leading to poor perfusion, thus raising the risk of preeclampsia, fetal growth restriction, and, in some cases, stillbirth. Furthermore, histamine release—often triggered by LPS stimulation and toxic microbial metabolites—along with further vasoactive inflammatory mediators like TNFα and IL6, may drive a multisystemic inflammatory state.

In the context of *Hp*-infection/MetS, mast cell-derived immunomodulatory cytokines, histamine, chymase, and metalloproteases are involved in leucocyte migration to inflammatory subendothelial areas, which are predisposed to atherogenesis, and display changed permeability (e.g., increased low-density lipoprotein infiltration), macrophage apoptosis, and vascular wall degradation. Similarly, disruptions in pericyte/endothelial signaling related to MetS/*Hp*-I leading to the development of placental vasculopathies further impede proper crosstalk at the maternal–fetal interface with subsequent pregnancy, delivery and post-partum abnormalities.

Under stress conditions, such as those often encountered in pregnancy, mast cell-released tryptase and histamine are released into the gut lumen, exacerbating gut penetrability and prolonging the inflammation. Augmented mucosal penetrability and a thinner mucous level facilitate interactions among mast cells, other immune cells, and the microbiota, sustaining a predominant Th1/Th17 shift paired with decreased tolerogenic Teg cell populations, increasing the odds for unfavorable outcomes such as preeclampsia, preterm labor, and fetal death.

#### 1.1.2. The Role of *H. pylori*/MetS-Related Hyperhomocysteinemia Pathologies and Their Potential Impact on Pregnancy and Neonatal Outcomes

The interactions between *H. pylori* and MetS-connected hyperhomocysteinemia are believed to be involved in atherosclerosis related to *H. pylori*/MetS, which is connected with systemic diseases like C-CVD and neurodegenerative conditions [2,53,54,55]. In particular, MetS combined with *H. pylori* infection-related chronic gastritis can lead to the malabsorption of vitamin B12 and folate. This malabsorption results in an ineffective methylation process by 5-methyl-tetrahydrofolic acid, subsequently causing an accumulation of homocysteine. As a proatherogenic factor, homocysteine independently elevates the risk of developing *H. pylori*/MetS-related C-CVD and additional systemic pathologies [54,56]. Hyperhomocysteinemia, hyperfibrinogenemia, and elevated levels of lipoprotein-a (a low-density lipoprotein-like particle that includes the plasminogen homologue apo (a) linked disulfide bound to apo B), are identified as “non-traditional” risk factors for CVD that might promote atherosclerosis and its related pathologies in the setting of *H. pylori*/MetS-related conditions [57].

Regarding pregnancy and neonatal outcomes related to hyperhomocysteinemia, recent evidence indicates that the prevalence of placenta-mediated pregnancy complications (PMPCs) is significantly high in women with hyperhomocysteinemia. Maternal hyperhomocysteinemia can be introduced as both a predictor of the development of PMPCs and a screening tool for low-risk antenatal patients in the early second trimester [57]. Hyperhomocysteinemia is closely connected with the risk of developing PMPCs such as preeclampsia, fetal growth restriction, intrauterine fetal death, preterm births, and placental abruption (Figure 1).

Women with hyperhomocysteinemia exhibit approximately a twelve-fold risk of preterm birth and a ten-fold risk of delivering a term neonate with low birth weight [58]. Moreover, fetal hyperhomocysteinemia during pregnancy is a possible risk factor that may initiate an early breakdown of uterine quiescence owing to oxidative stress and activation of inflammatory processes in the placenta leading to preterm birth [59]. Likewise, hyperhomocysteinemia is linked with preeclampsia and eclampsia, although, in eclampsia, the burden of hyperhomocysteinemia is more prominent than in preeclampsia [60].

A “cross-talk” of maternal–fetal homocysteine interrelationships describes the placental transport of homocysteine, its impact on pregnancy outcomes, and the effects of homocysteine and methylation on the risk of neural tube defects. It suggests a putative pathway of embryonic provision of folate and vitamin B12, which are nutrients that modulate homocysteine levels and ameliorate the risk of neural tube defects [61]. Higher homocysteine levels and lower folate concentration during early pregnancy are linked with adverse pregnancy and neonatal outcomes. Vitamin B12 deficiency is more frequent among pregnant women compared with folate deficiency. Hyperhomocysteinemia is an independent risk factor for pregnancy and neonatal complications, and vitamin B12 deficiency in the first and second trimesters is linked with offsprings’ low body weight [62,63]. Finally, experimental studies suggest a potential teratogenic effect of hyperhomocysteinemia and, owing to the high incidence of hyperhomocysteinemia in both the reproductive and general population, investigation into the underlying epigenetic mechanisms is needed [64].

Collectively, efforts to prevent atherosclerosis-related diseases and other systemic disorders, including those affecting pregnancy and newborn health, should focus on identifying and treating primarily *H. pylori* infection/MetS and hyperhomocysteinemia (regularly stemming from masked vitamin B12 deficit) [54,55]. Eradication of *H. pylori* in individuals with vitamin B12 deficit has been shown to increase vitamin B12 concentrations while simultaneously reducing homocysteine levels in the blood [65]. Additional studies are necessary to fully grasp the effects of *H. pylori* therapy in individuals with *H. pylori* infection and long-standing hyperhomocysteinemia [66]. Managing the effects of *H. pylori*/MetS-related hyperhomocysteinemia on systemic issues, including adverse pregnancy and neonatal events, could yield substantial health benefits. Further investigation is essential to fully elucidate this significant topic, which poses a significant worldwide challenge [5].

Interestingly, cardiac hypertrophy associated with hyperhomocysteinemia in rats is connected to oxidative stress and increased density of cardiac MC. Medications like sodium cromoglycate and ketotifen could potentially alleviate this pathology by decreasing oxidative stress and MC activation [67].

#### 1.1.3. The Role of *H. pylori*/MetS-Linked Galectin-3 Pathologies and Their Potential Impact on Pregnancy and Neonatal Outcomes

Galectin-3, linked with *H. pylori* and prevalent in conditions such as MetS and MAFLD [68,69], is further associated with a higher risk of all-cause mortality, particularly heart failure and C-CVD death [70]. *H. pylori*-associated galectin-3 overexpression and MetS appear to be implicated in the persistent and progressive dysfunction of various organs, including liver, CVD, kidney, and brain [71]. For example, galectin-3 plays a critical role in regulating the cerebral innate immune reactions, acting as an endogenic regulator of neuroinflammatory and neurodegenerative processes [71]. High circulating galectin-3 concentrations are significantly associated with the progression of both AD and Parkinson’s disease (PD) [72]. Additionally, higher galectin-3 levels are independently connected with depression in type 1 DM [73]. Type 1 DM is also associated with MetS [74,75] and *H. pylori* infection [76]. It is noteworthy that galectin-3, apart from its presence in other cell types, is also found in MCs, though the potential role of galectin-3-related MC activation in conditions such as C-CVD remains to be elucidated [71]. Specific inhibitors of galectin-3 inhibit microglial activation [77], representing a promising therapeutic target to curb neurodegenerative diseases and possibly other systemic disorders.

Regarding pregnancy and neonatal outcomes related to galectin-3, it is evident in all placental trophoblast cell lines, including villous cytotrophoblast cells and extravillous trophoblasts. Its abundance is inversely correlated with trophoblast invasiveness during the course of gestation, and the deregulation of placental galectin-3 is linked with obstetric complications, including spontaneous or repeated abortion [78,79]. Its abnormal expression is associated with obstetric complications such as preterm birth, preeclampsia, and fetal growth restriction [80] (Figure 1). Moreover, in preeclampsia, galectin-3 may contribute to the damaging effects of IR and dyslipidemia [81], which are also associated with MetS and *H. pylori* infection-related complications [18,29]. Maternal circulating galectin-3 concentrations are also considerably higher in pregnancies complicated with preterm prelabor rupture of membranes (PPROM). Galectin-3, with its regulatory effects in main biological processes, could be an initiating factor in the pathophysiology of PPROM, a predictive biomarker, and a target of preventing strategies of PPROM [82]. Additionally, umbilical cord plasma galectin-3 binding protein levels are increased in prematurity, possibly reflecting inflammatory processes in mother and infant [83]. Galectin-3 is also intensely expressed at molecular levels (mRNA and protein expression) in gestational diabetes mellitus maternal blood and placental tissue, suggesting a potential galectin-3 damaging effect. It is recognized that gestational diabetes mellitus raises the risk of adverse pregnancy and neonatal outcomes and long-term complications in both mothers and newborns [83]. Lastly, in intrahepatic cholestasis of pregnancy, a specific liver disorder typically emerging in the third trimester, elevated maternal serum and placental levels of galectin-3 suggest its involvement in the pathophysiology of this condition, pointing to galectin-3 as a potential initiator, diagnostic marker, and target for prevention strategies related to intrahepatic cholestasis of pregnancy [78].

#### 1.1.4. The Role of *H. pylori*/MetS-Related Atrial Fibrillation Pathologies and Their Potential Impact on Pregnancy and Neonatal Outcomes

Atrial fibrillation (AF) significantly contributes to the morbidity and mortality associated with *H. pylori*/MetS-related C-CVD [25], especially due to strokes caused by AF, which are similarly related to *H. pylori* infection/MetS [84]. AF is among the leading causes of stroke related to *H. pylori* infection and MetS [85]. There is also evidence suggesting a genetically predicted effect of AF on neurodegeneration due to ischemic stroke, thus identifying AF as a manageable risk factor for cognitive impairment and dementia following a stroke [86]. The link between *H. pylori*/MetS and the AF-related severity of CVD may involve various mediators, such as proinflammatory cytokines like TNFα and IL6, contributing to the development of atherosclerosis and AF-related complications. IR is also associated with AF and doubles the risk of C-CVD outcomes, including stroke [87]. Moreover, *H. pylori* infection/MetS may also contribute to the pathogenesis of MAFLD and its related AF adverse outcomes. Thus, *H. pylori* eradication may offer potential benefit for these pathological conditions, necessitating additional investigation [87,88].

Regarding *H. pylori*/Mets-related AF involvement in cerebral disorders, AF has been implicated in the progression from mild cognitive impairment (MCI) to dementia [89]. There is evidence connecting AF with an increased risk and mortality rate across all categories of dementias, such as vascular dementia and AD [90,91,92,93]. Catheter ablation in AF patients, beyond *H. pylori* eradication, appears to decrease the risk of dementia and AD [94]; AD is reduced in patients who have undergone AF catheter ablation [95].

Interestingly, there is evidence indicating that immune cells, such as MCs, could also contribute to AF pathophysiology [95,96]. Therefore, further studies are needed.

Regarding pregnancy-related AF, it is the most frequent arrhythmia in pregnancy, partly explained by increasing maternal age, cardiovascular risk factors, and congenital cardiac disease in pregnancy. AF is associated with adverse maternal and fetal/neonatal outcomes, including death [97]. Augmented maternal mortality and low fetal birth weight are observed in patients with AF during pregnancy (Figure 1), with an AF peak at the end of the second trimester [98]. AF causes significant morbidity in women and is typically attributed to cardiac remodeling from various causes, especially AH [99], which is similarly associated with diverse pathologies related to *H. pylori* infection/MetS [3,25,29]. Obstetric complications are often observed in individuals with AF. While the exact mechanism linking obstetric complications to AF remains unclear, it is plausible that conditions like preeclampsia could lead to increased adrenergic activity, heightened inflammatory response, and activation of the renin–angiotensin–aldosterone system. These factors may potentially induce electrophysiological changes in the atrium, increasing susceptibility to arrhythmias such as AF [100,101,102].

The connection between pregnancy-related problems and occurrences of AF may involve various mechanisms. Frequent risk issues, including MetS, may be involved in the pathophysiology of AF [103]. Additionally, pregnancy-related complications, which often involve cardiac remodeling and fibrosis, may result in long-term cardiac dysfunction [104,105,106], increasing the likelihood of AF onset. It is also possible that these complications heighten the risk of developing AF-related risk factors like AH, unfavorable lipid profiles, and T2DM [107,108,109]. Genetic factors may further influence these associations; for instance, single nucleotide polymorphisms (SNP) near the PITX2 gene are involved in both preeclampsia and AF [110]. Pregnancy creates a prothrombotic state, increasing the risk of stroke due to AF [111]; this heightened coagulability may elevate the risk of thromboembolic complications in pregnant women with AF [112]. Managing AF during pregnancy is crucial for maternal and fetal well-being. However, many medications commonly used to treat AF have been linked to adverse fetal outcomes [113], necessitating further research in this area.

#### 1.1.5. The Role of *H. pylori*/MetS-Related Gut Dysbiosis Pathologies and Their Potential Impact on Pregnancy and Neonatal Outcomes

Inflammatory processes, particularly those involving the nuclear factor κappa B (NF-κB) signaling pathway, are intimately linked with lipopolysaccharide (LPS) [114]. These inflammatory issues can lead to IR [115]. *H. pylori*-associated LPS has inflammatory activity, fostering the development of atherosclerosis and C-CVD. Gastrointestinal bacteria like *H. pylori* can trigger inflammation associated with MetS parameters through LPS activation, showing a potential link among LPS strong activity and the occurrence of MetS activity. This bacterium has also been connected to gut dysbiosis [116], and dysbiosis in the microbiota associated with *H. pylori*/MetS may be involved in the pathophysiology of MAFLD [18] and its negative outcomes, including stroke and neurodegeneration. Conversely, transplanted fecal material has shown potential in inhibiting MAFLD and ameliorating cerebral injury-induced dysbiosis, thus improving outcomes in disorders like stroke [18]. Additionally, probiotics, symbiotics, and postbiotics have been used to manage neurodegenerative diseases such as AD by modulating intestinal dysbiosis [117].

Gut dysbiosis emerges as a significant factor linked to *H. pylori*/MetS, contributing to the pathophysiology of age-related atherosclerosis, T2DM, and neurodegenerative pathologies [118,119]. Dysbiosis, by increasing intestinal permeability, can facilitate the translocation of bacterial products like LPS into circulation, which can access the brain and contribute to neurodegeneration [114,120]. This condition, primarily triggered by dysbiosis, is closely associated with the development and progression of various *H. pylori*/MetS-related pathologies, such as MAFLD, C-CVD, AD, MCI, PD, multiple sclerosis (MS), and glaucoma (termed as “ocular AD”) [4,121,122].

Considering pregnancy related-gut dysbiosis (Figure 1), it has been associated with pregnancy complications and negative fetal/neonatal outcomes [123]. Gut dysbiosis serves as a potential modulator of antenatal disorders related to the placenta, including fetal growth restriction, preeclampsia, maternal obesity, gestational diabetes mellitus, and preterm deliveries [123,124]. In this regard, a potential relationship between gestational diabetes mellitus and gut dysbiosis occurs in mothers and newborns, and there are influencing factors derived from gestational diabetes mellitus mothers on the gut dysbiosis of their newborns, including the vertical transmission of microbiota from mothers [125]. Furthermore, elevated levels of plasma LPS have been observed in patients with preeclampsia, and the gut dysbiosis linked with the LPS synthesis along with augmented placental LPS concentrations are also described [126]. This suggests that gut dysbiosis may manifest from the early stages of preeclampsia development, implicating potential etiological and therapeutic implications [127]. Additional research conducted in antibiotic-treated mice colonized with fecal microbiota from fetal growth restriction has corroborated previous findings of gut dysbiosis in fetal growth restriction and demonstrated that maternal gut dysbiosis contributes to placental impairment [128]. The movement of microorganisms or their products from the intestine to the placenta could lead to alterations in the placental tissue and function under pathological states, proposing the gut–placenta axis as a key factor in the etiology of preeclampsia [129]. Importantly, maternal dysbiosis could contribute to the growth of disorders in adulthood, including metabolic CVD, neurodevelopment, and/or immune system alterations [123].

Finally, many data indicate that probiotics, prebiotics, and synbiotics confer health benefits in preventing adverse pregnancy and neonatal outcomes; the use of probiotics and prebiotics during pregnancy appears to mitigate adverse outcomes [130].

#### 1.1.6. The Role of MetS and *H. pylori*-Related MC Activation Pathologies and Their Potential Impact on Pregnancy and Neonatal Outcomes

MCs, initially identified for their impact on allergic and anaphylactic conditions, are now considered important contributors to the complex mechanisms underlying MetS [131]. Located within adipose tissue, MCs are central to the chronic inflammatory processes associated with obesity, a fundamental aspect of MetS [132].

Activation of MCs provokes the enrolment of immune cells, including lipid-accumulating foamy macrophages, thereby intensifying inflammatory reaction and promoting angiogenesis [133]. This immune cell infiltration significantly contributes to the perpetuation of the MetS phenotype [134].

MetS is closely linked with MC activation [135]. The mediators released by MCs contribute to IR, thereby promoting the development of atherosclerosis [135], a condition strongly associated with MetS [136]. The correlation among atherosclerosis and MetS is multifactorial, involving MetS-related parameters like IR as substantial contributors to its onset and progression [137].

Specifically, MCs could play a pivotal role in the pathophysiology of *H. pylori*/MetS-related conditions, including hyperglycemia, obesity, dyslipidemia, IR, T2DM, cardio-metabolic conditions, AD, referred to as “type-3 diabetes”, and their complications [135,138,139,140]. For example, dyslipidemia is strongly linked with *H. pylori*/MetS, and MC activation, through the release of tryptase and chymase, contributes to dyslipidemia [141], while eradication of *H. pylori* improves lipid profiles such as fibrinogen, an independent risk factor for MetS-related C-CVD and dementia [25,142].

*H. pylori*, by activating MCs, disrupts the balance of gut microbiota and may be involved in MetS-related local and systemic disorders [142].

Locally, *H. pylori*-related IL33 appears to orchestrate MC responses and promote bacterial expansion, thus implicating the induction of gastritis [142]. Patients with *H. pylori* display high numbers of MCs in the gastric mucosa and apoptotic cells [143]. In addition to IL33, MC chymase may be another significant contributor to *H. pylori*-induced gastritis [144].

Systemically, *H. pylori*’s virulence factor neutrophil-activating protein [145] activates MCs, among others, leading to the secretion of proinflammatory mediators [146]. Moreover, *H. pylori* and MC-related atherosclerosis may contribute to MetS-related conditions like T2DM [147]. Remarkably, the presence of *H. pylori* DNA in atherosclerotic lesions and the association of this infection with the onset of carotid plaque in adults without previous C-CVD imply that *H. pylori* infection may be involved in the pathophysiology of atherosclerotic systemic diseases [148].

Regarding MC activation and its impact on adverse pregnancy outcomes such as preeclampsia, these cells, as mentioned before [8], exhibit adverse effects throughout pregnancy and the post-partum period (Figure 1) [27,149]. MCs may contribute to fetal growth restriction and stillbirth [150]. As an additional example, while human MCs can increase the biological behaviors of trophoblasts to establish pregnancy, human MC corticosteroid therapy constrains this process, thereby leading to infertility [151].

## 2. Conclusions

Taken altogether, parameters related to *H. pylori*/MetS, including pericyte dysfunction, hyperhomocysteinemia, galectin-3, AF, and/or gut dysbiosis, may play roles in negative pregnancy and neonatal outcomes. Equally, *H. pylori*/MetS-related MC activation may also be involved in adverse pregnancy and neonatal outcomes. Therefore, further studies are mandatory to elucidate this critical topic in depths thereby offering related therapeutic strategies to mitigate adverse outcomes for mother, fetuses, and neonates, representing a top global health issue.

## Data Availability

Not applicable.

## Abbreviations

AF: atrial fibrillation; DC: dendritic cell; E2: estradiol; GDM: gestational diabetes mellitus; *Hp*-I: *Helicobacter pylori* infection; IL: interleukin; IFN: interferon; IR: insulin resistance; LPS: lipopolysaccharide; MetS: metabolic syndrome; PAMPs: pathogen-associated molecular patterns; PRG: progesterone; PPROM: preterm prelabor rupture of membranes; SCFAs: short-chain fatty acids; TNFα: tumor necrosis factor-α; TMAO: trimethylamine N-oxide; TLR-: toll-like receptor; VacA: vacuolating cytotoxin.
